# Supplementary material for: A New Albomycin-Producing Strain of Streptomyces globisporus subsp. globisporus May Provide Protection for Ants Messor structor
Source: Insects. 2022 Nov 11;13(11):1042. doi: 10.3390/insects13111042 (PMC9693239; doi:10.3390/insects13111042)
Supplement: Supplementary file 1 [file insects-13-01042-s001.zip › insects-1991976-supplementary.pdf]

insects

MDPI

A New Albomycin-Producing Strain of *Streptomyces globisporus* subsp. *globisporus* May Provide Protection for Ants *Messor structor*

Supplementary Materials

Yuliya V. Zakalyukina [1,2,*], Nikolay A. Pavlov [3], Dmitrii A. Lukianov [3,4], Valeria I. Marina [3,4], Olga A. Belozerova [5], Vadim N. Tashlitsky [4], Elena B. Guglya [5,6], Ilya A. Osterman [1,3,4] and Mikhail V. Biryukov [1,3,7]

1 Center for Translational Medicine, Sirius University of Science and Technology, Olympic Avenue 1, 354340 Sochi, Russia
2 Department of Soil Science, Lomonosov Moscow State University, Leninskie Gory 1, 119991 Moscow, Russia
3 Center of Life Sciences, Skolkovo Institute of Science and Technology, Bolshoy Boulevard 30, Bld. 1, 121205 Moscow, Russia
4 Department of Chemistry, Lomonosov Moscow State University, Leninskie Gory 1, 119991 Moscow, Russia
5 Shemyakin-Ovchinnikov Institute of Bioorganic Chemistry of the Russian Academy of Sciences, Miklukho-Maklaya st. 16/10, 117997 Moscow, Russia
6 Institute of Translational Medicine, Pirogov Russian National Research Medical University, Ostrovityanova st. 1, Moscow 117997, Russia
7 Department of Biology, Lomonosov Moscow State University, Leninskie Gory 1, 119991 Moscow, Russia
* Correspondence: juline@soil.msu.ru (Y.V.Z.); Tel.: +7-9175548004

Content

Table S1. The isolation of Gram-positive bacteria strains from *Messor structor* individuals 3

Table S2. General features of the genome of strain 4-3 and its closely related strains of *Streptomyces globisporus* 4

Figure S1. Neighbour-joining phylogenetic tree of strain 4-3, related *Streptomyces globisporus* subsp. *globisporus* and some actinobacteria species, firstly isolated from insects 5

Figure S2. Maximum-parsimony phylogenetic tree based on almost complete 16S rRNA gene sequences (1445 positions in the final dataset) showing the position of 4-3 among the related strains of the genus *Streptomyces* and type actinobacterial species isolated from insects 6

Figure S3. Maximum-likelihood phylogenetic tree of strain 4-3 and comparison with related strains of the genus *Streptomyces* and type actinobacterial species isolated from insects, based on almost complete 16S rRNA gene sequences 7

Table S3. Morphological, physiological and biochemical characteristics of strain 4-3 and closest *Streptomyces globisporus* subsp. *globisporus* strains 8

Figure S4. Cultural properties of 4-3 on ISP media after 14 days at 28℃ 9

Figure S5. Utilization of different sugars (1.0%, w/v) as sole carbon source by strain 4-3 10

Figure S6. RP HPLC of an active fraction and UV spectrum of an active metabolite peak 11

Figure S7. LC-MS data for albomycin δ2 12

Table S4. Some of the secondary metabolite gene clusters in *Streptomyces globisporus* subsp. *globisporus* 4-3 13

Table S5. Antimicrobial activity of *Streptomyces globisporus* subsp. *globisporus* 4-3 14

Table S1. The isolation of Gram-positive bacteria strains from *Messor structor* individuals

Caste	Isolated strains	Frequency of occurrence[1],%	Identified as
Solders	M1, S1, S3, S4, S5, S51, Mdom1, Mdom2, Mdom3 (4-3)	89	*Streptomyces globisporus* subsp. *globisporus* strain 4-3[2]
	Am1, Am2, Mr	44	*Staphylococcus gallinarum* L1[3]
Workers	M1, S1, S3, S4, S5, S51, Mdom1, Mdom2	50	*Streptomyces globisporus* subsp. *globisporus* strain 4-3
	Am1, Am2, Mr	88	*Staphylococcus gallinarum* L1
Larvae	MdomX1	7	*Streptomyces globisporus* subsp. *globisporus* strain 4-3
	L1	85	*Staphylococcus gallinarum* L1
Pupae	MdomX1, MdomX2	21	*Streptomyces globisporus* subsp. *globisporus* strain 4-3
	L1	85	*Staphylococcus gallinarum* L1

[1] -- the proportion of individuals of the studied caste in which this strain was isolated,
[2] -- GenBank accession number for 16S rRNA gene sequence is ON326616,
[3] -- GenBank accession number is ON326604

Table S2. General Neighbour-joining phylogenetic tree strain 4-3 and its closely related strains of *Streptomyces globisporus*

Genomic features	1	2	3	4	5
Size (Mbp)	7,941,828	8,246,570	7,237,318	7,548,572	7,332,794
Total gene	7,449	7,410	6,729	6,822	6,762
Contigs	139	59	594	54	51
G +C content (%)	71.6	71.4	71.4	71.7	71.7
No. of rRNA clusters	3	1	5	3	5
No. of tRNA clusters	65	68	63	65	63
Contig N50	375,468	434,455	22,344	268,800	459,850
Contig L50	9	6	100	7	5
No. proteins	7,142	7,495	6,258	6,622	6,593
Completeness of genome*, %	99.1	99.1	95.3	99.1	97.2
Quality of genome*, %	56.6	47.1	62.3	52.1	54.7

Strains, (GenBank assembly accessions are indicated in parentheses): 1, 4-3 (SAMEA13759927); 2, *Streptomyces globisporus subsp. globisporus* DSM 40199^T (GCA_014649555.1); 3, *Streptomyces globisporus subsp. globisporus* DSM 40136 (GCA_002154385.1); 4, *Streptomyces rubiginosohelvolus* DSM 40176^T (GCA_014649875.1); 5, *Streptomyces pluricolorescens* DSM 40019^T (GCA_014650395.1).

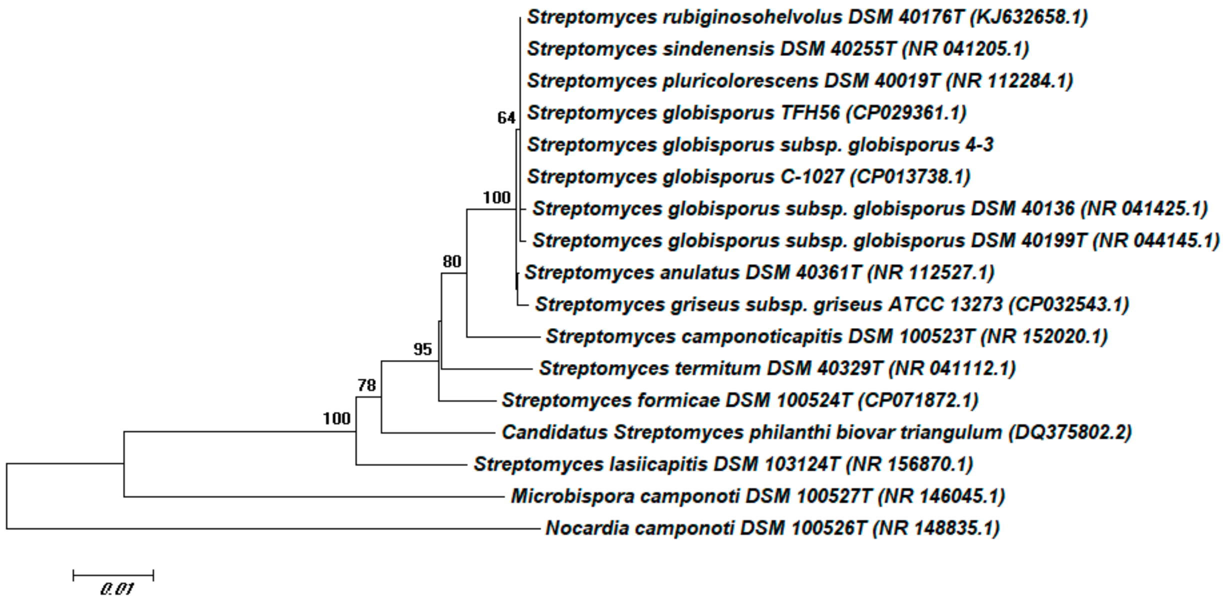

Figure S1. Neighbour-joining phylogenetic tree of strain 4-3, related *Streptomyces globisporus* subsp. *globisporus* and some actinobacteria species, firstly isolated from insects. The evolutionary distances were computed from 16S rRNA full-length sequences using the Tamura-Nei method. Bootstrap values for n=1,000 are given at the branching points in percentages for values higher than 60. Bar, 0,01 nucleotide substitutions per site. The analysis involved 17 nucleotide sequences with a total of 1472 positions in the final dataset. *Nocardia camponoti* DSM 100526[T] was used as the outgroup.

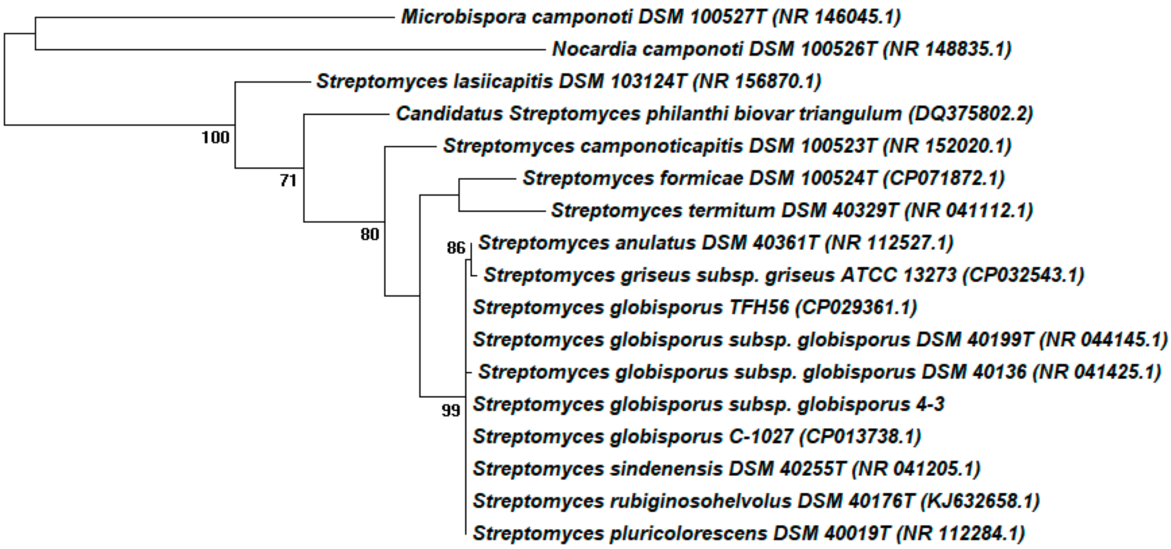

Figure S2. Maximum-parsimony phylogenetic tree based on almost complete 16S rRNA gene sequences (1445 positions in the final dataset) showing the position of 4-3 among the related strains of the genus *Streptomyces* and type actinobacterial species isolated from insects. Tree #1 out of 2 most parsimonious trees (length = 353) is shown. The consistency index is (0,661765), the retention index is (0,634921), and the composite index is 0,510814 (0,420168) for all sites and parsimony-informative sites. The percentage of replicate trees in which the associated taxa clustered together in the bootstrap test (1000 replicates) are shown next to the branches. The MP tree was obtained using the Subtree-Pruning-Regrafting (SPR) algorithm with search level 1 in which the initial trees were obtained by the random addition of sequences (10 replicates). The tree is drawn to scale , with branch lengths calculated using the average pathway method and are in the units of the number of changes over the whole sequence. Codon positions included were 1st+2nd+3rd+Noncoding. All positions containing gaps and missing data were eliminated.

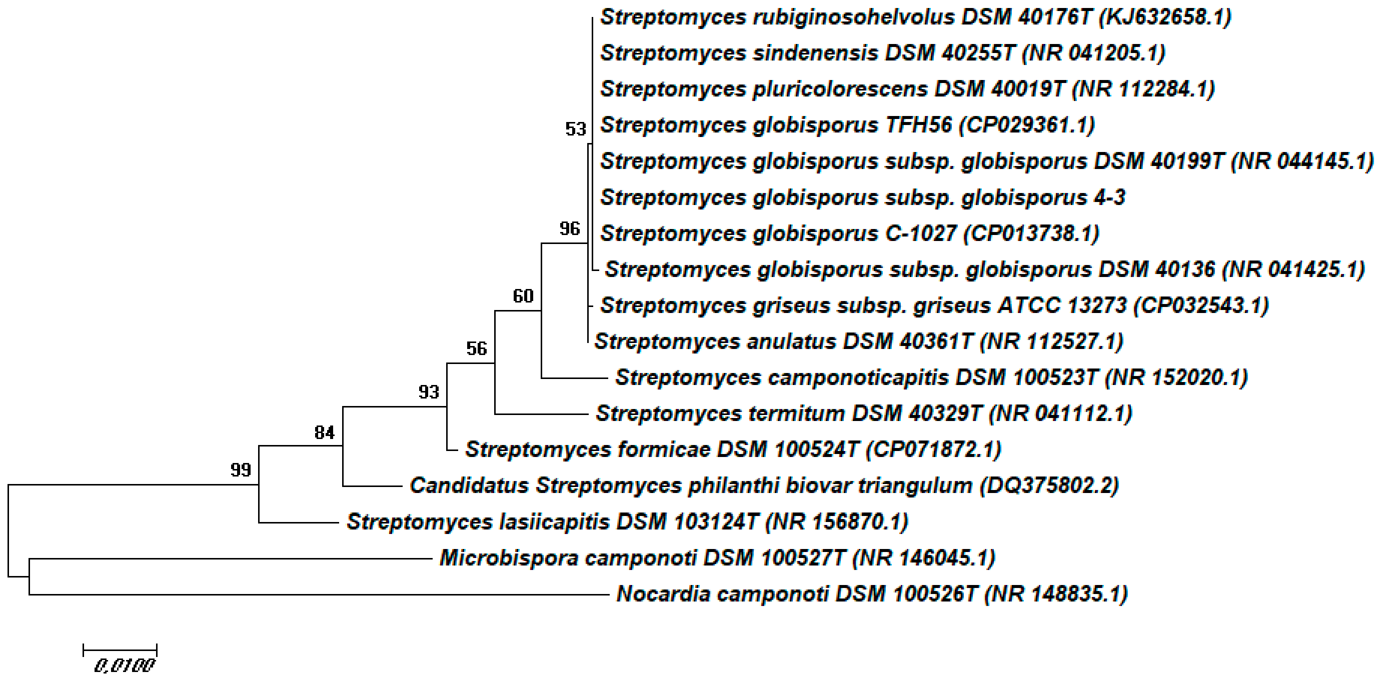

Figure S3. Maximum-likelihood phylogenetic tree of strain 4-3 and comparison with related strains of the genus *Streptomyces* and type actinobacterial species isolated from insects, based on almost complete 16S rRNA gene sequences. Maximum Likelihood method based on the Tamura-Nei model. The tree with the highest log likelihood (-3854,6731) is shown. The percentage of trees in which the associated taxa clustered together is shown next to the branches. Initial tree(s) for the heuristic search were obtained automatically by applying Neighbor-Join and BioNJ algorithms to a matrix of pairwise distances estimated using the Maximum Composite Likelihood (MCL) approach, and then selecting the topology with superior log likelihood value. The tree is drawn to scale, with branch lengths measured in the number of substitutions per site. The analysis involved 17 nucleotide sequences. Codon positions included were 1st+2nd+3rd+Noncoding. All positions containing gaps and missing data were eliminated. There were a total of 1445 positions in the final dataset.

Table S3. Morphological, physiological and biochemical characteristics of strain 4-3 and closest *Streptomyces globisporus* subsp. *globisporus* strains

**Properties**	**1**	**2[1]**	**3[1]**
Morphological and physiological characteristics			
Spore surface	Smooth	Smooth	Smooth
Spore chains morphology	RF	RF	RF
Temperature-range and optimum (°C)	8-37 (28)	(28)	(28)
NaCl tolerance (%)	5	n/d[2]	n/d
Acid from carbohydrates			
Arabinose	+	+	+
Dulcite	-	n/d	n/d
Fructose	+	+	+
Galactose	+	n/d	n/d
Glucose	+	-	+
Inositol	-	-	-
Lactose	-	n/d	n/d
Maltose	+	n/d	n/d
Mannitol	+	+	+
Raffinose	-	-	-
Rhamnose	+	+	+
Sorbitol	-	n/d	n/d
Sucrose	-	-	-
Xylose	+	-	-
Degradation of and enzymes			
Starch	+	+	+
Gelatin	+	+	+
Cellulose	-	+	+
Urea	+	+	+
Citrate	+	+	+
β-glucosidase	+	+	+
L-ornithine decarboxylase	-	+	+
L-arginine decarboxylase	-	+	+
L-lysine decarboxylase	+	+	+

[1] Data for *Streptomyces globisporus* subsp. *globisporus* DSM 40199^T (2) and *Streptomyces globisporus* subsp. *globisporus* DSM 40136^T (3) are from https://bacdive.dsmz.de/
[2] n/d, not determined

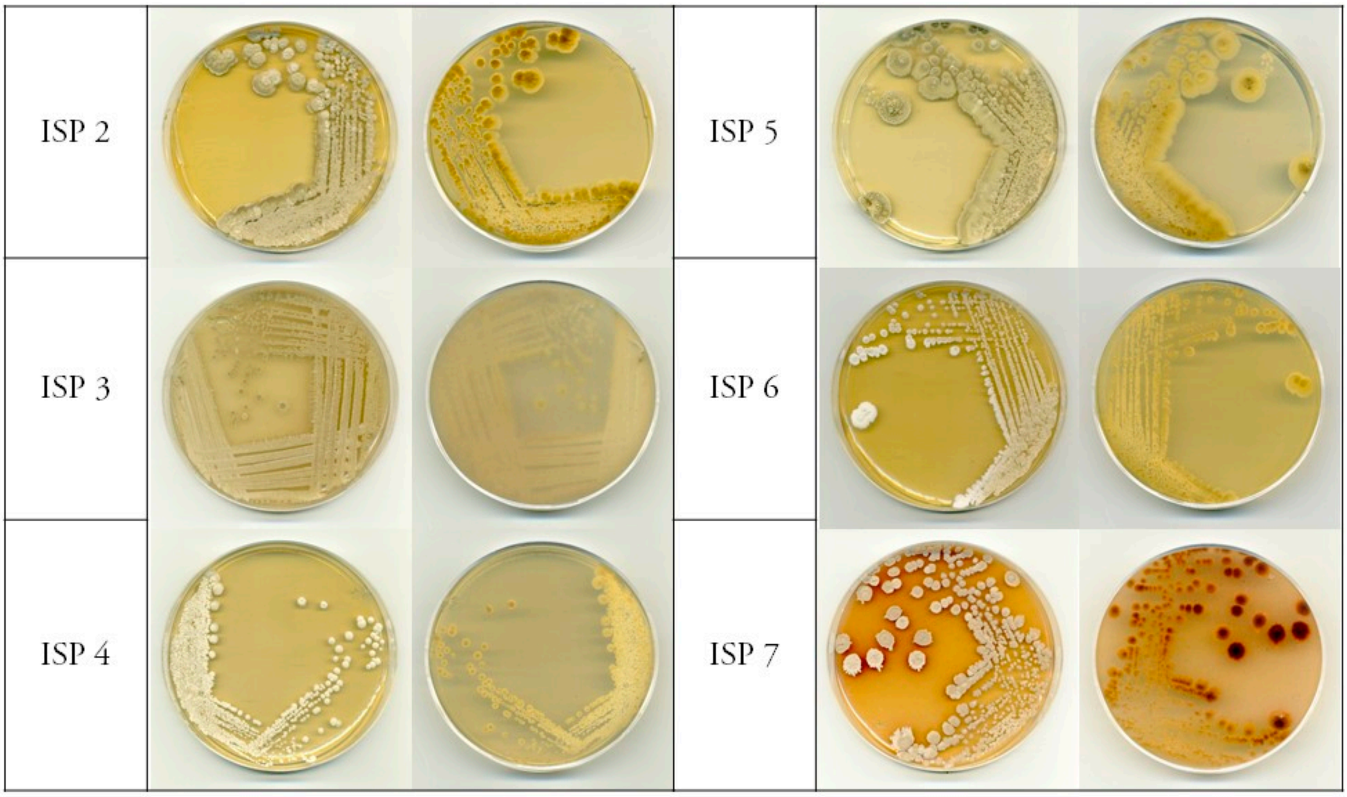

Figure S4. Cultural properties of 4-3 on ISP media after 14 days at 28℃.

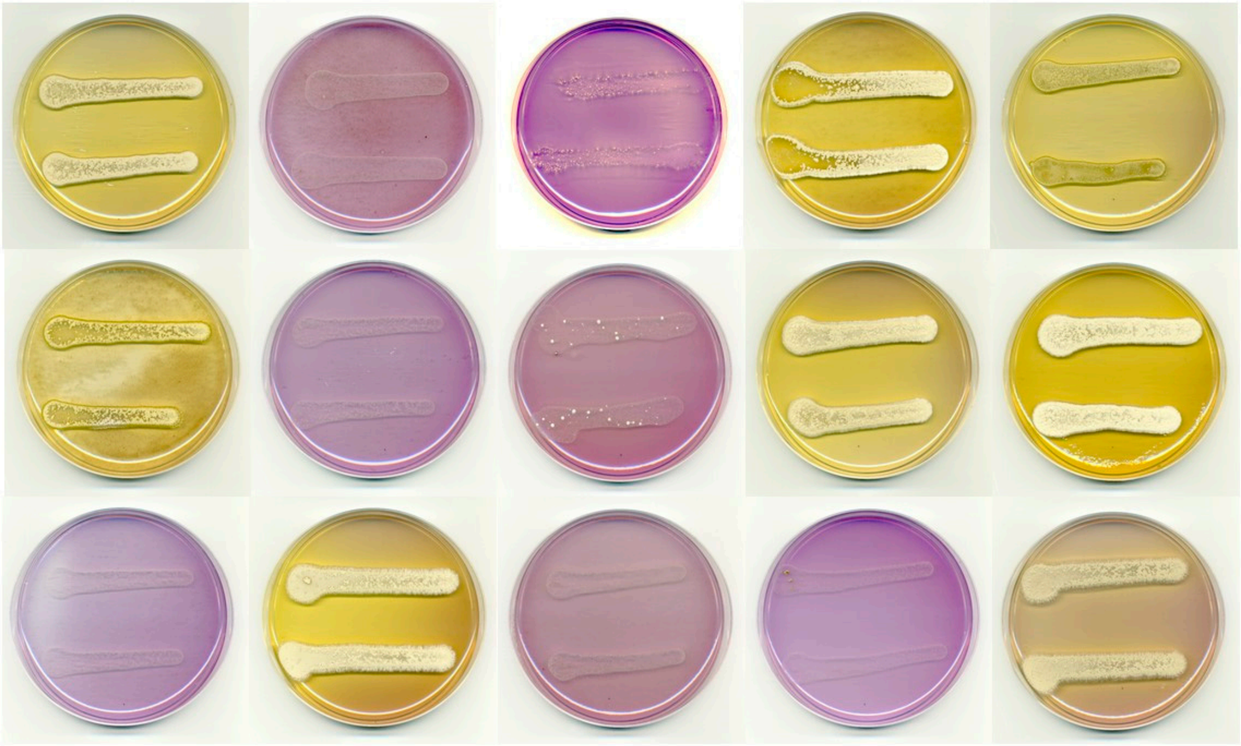

Figure S5. Utilization of different sugars (1.0%, w/v) as sole carbon source by strain 4-3.

Top row: arabinose, base agar without sugar, cellulose, fructose, galactose;

middle row: glucose, inositol, lactose, mannitol, mannose;

bottom row: raffinose, rhamnose, sorbitol, sucrose, xylose.

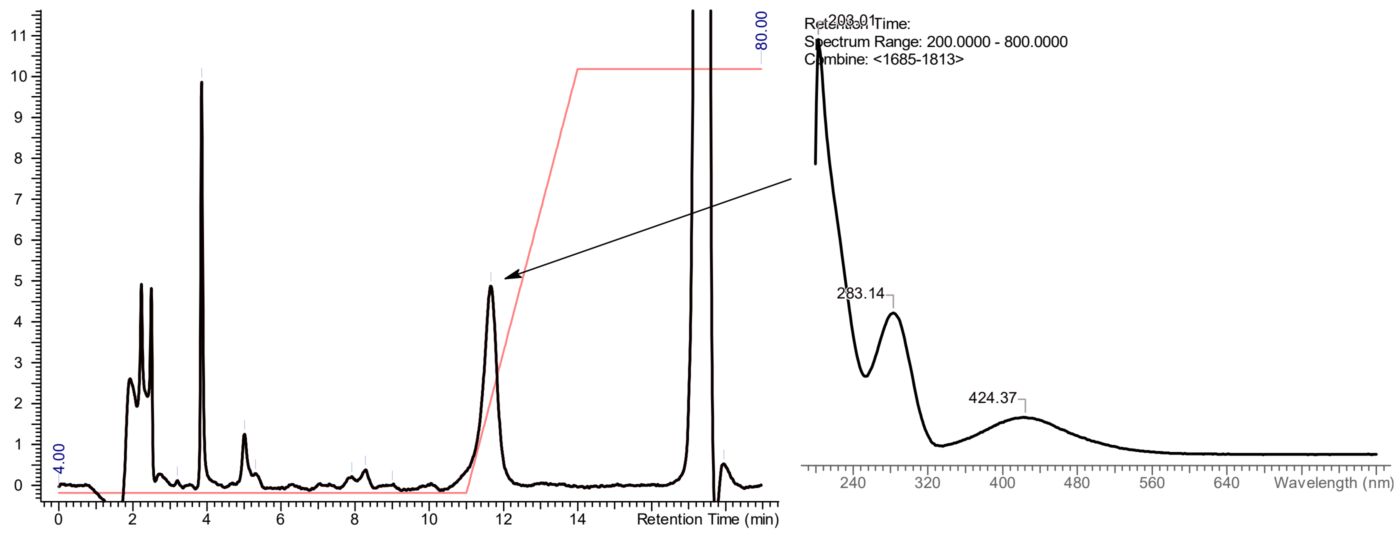

Figure S6. RP HPLC of an active fraction of culture liquid eluted from the LPS500H sorbent and UV spectrum of an active metabolite peak at 11.5 min. Column: Phenomenex Luna 5 µm C18 (2) 100 Å 4.6*250 mm; eluent: 4% of MeCN, 10 mM AcONH4, Flow rate 1ml/min, Temperature 25C, UV260 nm

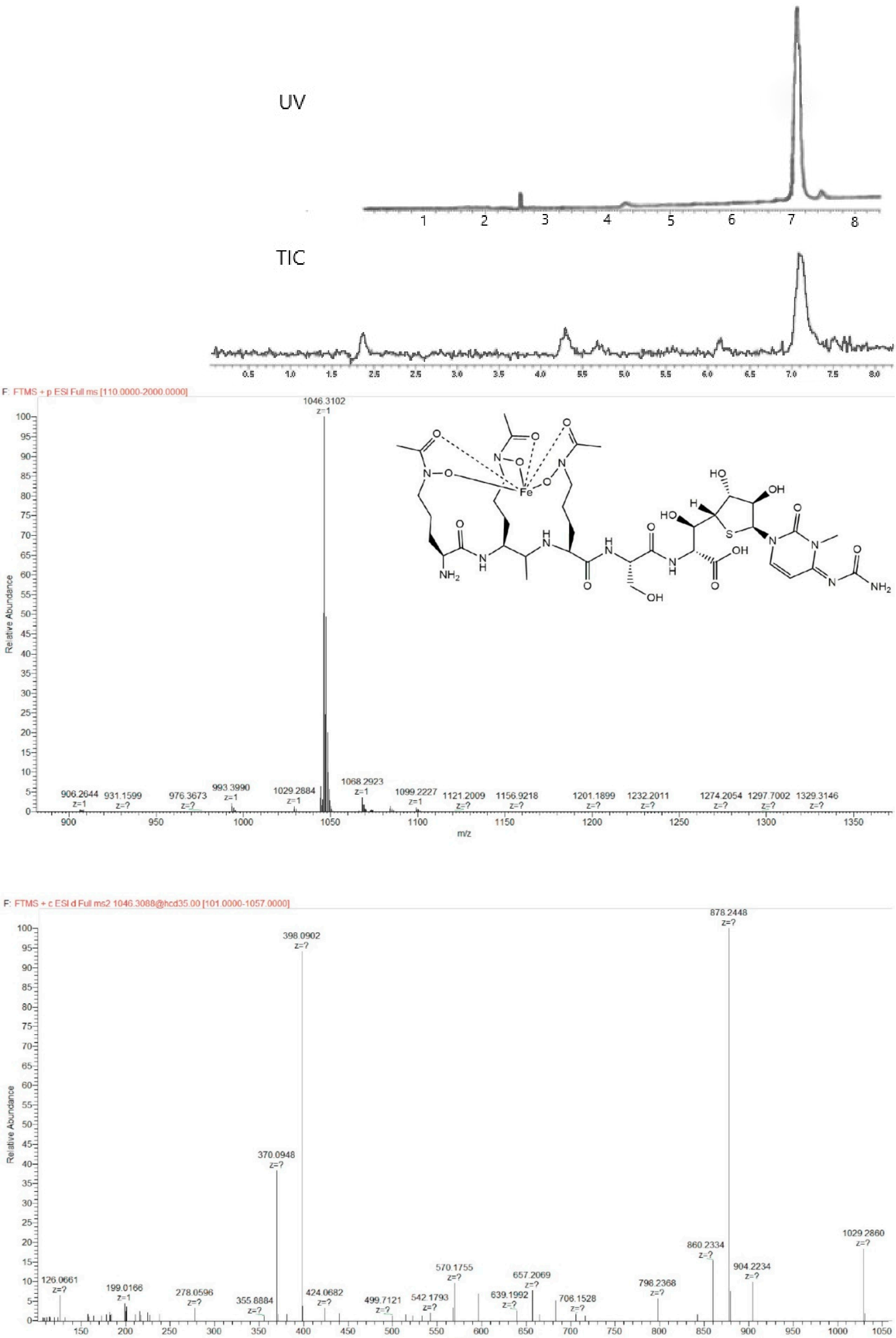

Figure S7. LC-MS data for albomycin δ2 (top down): UV profile at 287 nm; TIC collected at positive ion mode, MS1 and MS2 for the peak at 7.2 min.

Table S4. Some of the secondary metabolite gene clusters in *Streptomyces globisporus* subsp. *globisporus* 4-3

Region	Type	From (bp)	To (bp)	Most similar known cluster	Similarity, %
1.1	NRP	287,765	324,504	Streptophenazines B/C/E/H/G biosynthesis gene cluster	100
	Polyketide	324,738	347,987	Mayamycin biosynthesis gene cluster	100
2.1	RiPP:Lanthipeptide	58,226	81,129	Labyrinthopeptins A2/A1/A3 biosynthetic gene cluster	40
4.1	Polyketide	277,202	399,632	Viguiepinol biosynthetic gene cluster	73
7.2	Siderophore	84,661	96,439	Desferrioxamine B biosynthetic gene cluster	100
8.1	Terpene	301,258	327,831	Hopene biosynthetic gene cluster	69
9.1	RiPP: Lassopeptide	350,361	373,055	Keywimysin biosynthetic gene cluster	100
10.1	Other: Melanin	81,003	91,476	Melanin biosynthetic gene cluster	100
10.2	Polyketide	129,829	170,881	Alkylresorcinol biosynthetic gene cluster	100
14.1	Terpene	23,415	48,421	Isorenieratene biosynthetic gene cluster	100
14.3	Terpene	128,324	174,416	Geosmin biosynthetic gene cluster	100
14.4	NRPS: Siderophore	211,154	258,557	Streptobactin biosynthetic gene cluster	100
21.1	RiPP:Lanthipeptide	148,549	166,688	Lanthipeptide(s) Putative Class III	100
24.1	NRPS: Siderophore	1	36,289	Coelichelin biosynthetic gene cluster	81

Table S5. Antimicrobial activity of *Streptomyces globisporus* subsp. *globisporus* 4-3, isolated from *Messor structor* ants

Test organisms	Zones of growth inhibition*, mm	MIC, ng/µl
E.coli BW25113ΔtolC	7±0.9	0.375
Bacillus subtilis ATCC 6633	5±0.6	n/a
Staphylococcus aureus ATCC 25923	10±2.0	n/a
Aspergillus niger INA 00760	5±0.8	n/a
Candida albicans CBS 8836	0	n/a
Paenibacillus alvei VKM B-502	20±1.6	1.5
Bacillus thuringiensis VKM B-6650	15±2.4	3.0
Beauveria bassiana VKM F-1357	12±1.4	n/a
Entomophthora coronata VKM F-1359	15±3.6	n/a

* – Data are means ± standard error over five replicates.
